# Prevalence and Molecular Characterization of the Hepatitis E Virus in Retail Pork Products Marketed in Canada

**DOI:** 10.1007/s12560-017-9281-9

**Published:** 2017-02-14

**Authors:** Oksana Mykytczuk, Jennifer Harlow, Sabah Bidawid, Nathalie Corneau, Neda Nasheri

**Affiliations:** National Food Virology Reference Centre, Bureau of Microbial Hazards, Food Directorate, Health Canada, 251 Sir Frederick Banting Driveway, Ottawa, ON K1A 0K9 Canada

**Keywords:** Hepatitis E virus, Pork products, Droplet digital PCR, Phylogenetic analysis, Molecular detection

## Abstract

Infection with the hepatitis E virus (HEV) is very common worldwide. HEV causes acute viral hepatitis with approximately 20 million cases per year. While HEV genotypes 1 and 2 cause large waterborne and foodborne outbreaks with a significant mortality in developing countries, genotypes 3 and 4 are more prevalent in developed countries with transmission being mostly zoonotic. In North America and Europe, HEV has been increasingly detected in swine, and exposure to pigs and pork products is considered to be the primary source of infection. Therefore we set out to investigate the prevalence of HEV in retail pork products available in Canada, by screening meal-size portions of pork pâtés, raw pork sausages, and raw pork livers. The presence of the HEV genomes was determined by RT-PCR and viral RNA was quantified by digital droplet PCR. Overall, HEV was detected in 47% of the sampled pork pâtés and 10.5% of the sampled raw pork livers, but not in the sampled pork sausages, and sequencing confirmed that all HEV strains belonged to genotype 3. Further phylogenetic analysis revealed that except for one isolate that clusters with subtype 3d, all isolates belong to subtype 3a. Amino acid variations between the isolates were also observed in the sequenced capsid region. In conclusion, the prevalence of HEV in pâtés and raw pork livers observed in this study is in agreement with the current HEV distribution in pork products reported in other developed countries.

**Electronic supplementary material:**

The online version of this article (doi:10.1007/s12560-017-9281-9) contains supplementary material, which is available to authorized users.

## Introduction

Foodborne transmission of hepatitis E virus (HEV) is a public health issue of increasing importance in developed countries (Colson et al. 2012; Pavio et al. 2015; Sayed et al. 2015; Wilhelm et al. 2015). While in developing countries HEV causes large epidemics, sporadic cases of locally acquired HEV infections are increasingly reported in developed countries (Teshale et al. 2010). Although the epidemiological significance of HEV infections in industrialized countries requires further study, the zoonotic transmission of HEV via the consumption of raw or undercooked pork, wild boar, and deer meat has been confirmed by several studies (Bouamra et al. 2014; Colson et al. 2010; Masuda et al. 2005; Renou et al. 2014; Yapa et al. 2016). HEV typically causes self-limiting, acute hepatitis with a low mortality rate of 1–4%, except for pregnant woman in endemic regions where the mortality rate can be up to 20% (Purcell and Emerson 2008). Recently, an increasing number of chronic HEV infections that rapidly progress to cirrhosis has been reported in immunosuppressed patients (Kamar et al. 2014, 2015; Murali et al. 2015). Furthermore, extra-hepatic manifestations, such as neurological disorders, have been observed in immunocompetent HEV-infected patients (Abravanel et al. 2014; Dalton et al. 2015; Kamar et al. 2015; Koning et al. 2015).

HEV is a single-stranded, positive-sense RNA virus that belongs to the *Hepeviridae* family. Its genome is approximately 7.2 kb long and contains three overlapping open reading frames (ORF1, ORF2, and ORF3). ORF1 encodes non-structural proteins, including the viral replicase proteins. ORF2 encodes a 660-amino acid viral capsid that is the most immunogenic protein expressed by HEV and is responsible for the induction of immune responses. ORF3 encodes a small multifunctional protein (Cao and Meng 2012; Fujiwara et al. 2014).

Based on sequence variability in the full-length genome of different strains, HEV has been classified into four major genotypes that infect humans (HEV-1, HEV-2, HEV-3, and HEV-4) and several other genotypes that infect a wide range of vertebrates (Debing et al. 2016; Panda and Varma 2013; Smith et al. 2014; Wang et al. 2014). HEV-1 and HEV-2 that infect humans and non-human primates are responsible for large waterborne epidemics in subtropical and tropical regions. Also they cause high mortality in pregnant women and young children (Purcell and Emerson 2008; Teshale et al. 2010). In contrast, the main host for HEV-3 and HEV-4 is swine but these viruses can also infect humans and cause sporadic infections, as well as outbreaks in developed countries (Pavio et al. 2015; Perrin et al. 2015; Purcell and Emerson 2008). An increasing number of locally acquired human HEV-3 infections are reported in developed countries with diverse clinical manifestations. The pathogenicity of HEV-3 is particularly enigmatic since both immunocompetent and immunocompromised individuals can become infected, with or without extra-hepatic manifestations, while some HEV-seropositive individuals remain asymptomatic (Garbuglia et al. 2015; Kamar et al. 2015; Koot et al. 2015; Perrin et al. 2015; Teshale et al. 2010). Although foodborne transmission of HEV-3 and HEV-4 contributes to the spread of HEV infection, the public health risks associated with the consumption of contaminated retail meat are yet to be determined (Murali et al. 2015; Pavio et al. 2015). In Canada, several cases of locally acquired chronic HEV infections have been reported in liver-transplant and bone marrow-transplant patients in the province of Québec (Halac et al. 2012a, b). Due to the high sequence similarity between the HEV-3 in these patients and the strains found in pig farms in Québec, zoonotic transmission is suspected. Importantly, Yoo and colleagues have estimated that the seroprevalence of HEV in commercial pigs is as high as 88.8% in Québec, 80.1% in Ontario, and about 60% Canada-wide (Pei and Yoo 2002). Additionally, the HEV genome has been recently detected in Canadian retail pork chops and pork livers, as well as in the pork production chain (Nantel-Fortier et al. 2016; Wilhelm et al. 2014). Therefore, there is an urgent need for more research to identify other potential transmission sources that may pose HEV infection risk.

In order to have a realistic understanding of HEV exposure levels to the consumers, we screened meal-size portions (Canadian Community Health Survey, Cycle 2.2 2004) of retail pork pâté, raw pork sausages, and raw pork livers for the presence of HEV genome. For this purpose, we applied a viral extraction method involving sequential filtration of food homogenates to remove PCR inhibitors, and performed conventional RT-PCR for the initial detection of the HEV genome (Martin-Latil et al. 2014) and droplet digital PCR for quantification of the HEV viral load in positive samples. We also performed Sanger sequencing for genotype determination and phylogenetic analysis.

## Methods and Materials

### Sample Collection

Various brands of pork pâté, raw pork sausages, and raw pig livers were collected monthly from local grocery stores in the Ottawa region between March 2014 and September 2015. A total of 76 pâtés, 19 livers, and 35 raw sausages were chosen from either the same lot, different lots, or from packages prepared in-store without lot numbers. Samples were stored at 4 °C for short term or −20 °C for long term until they were processed.

### Preparation of Internal Sample Process Controls

FCV was propagated in Crandell Rees feline kidney (CRFK) cells as previously described (Bidawid et al. 2003). HAV was propagated in fetal rhesus monkey kidney (FRhK-4) cells as previously described (Bidawid et al. 2000). HAV and FCV stocks were obtained after three cycles of cell lysis by freezing and thawing at −80 °C and centrifugation at 1500×*g* for 15 min. Dead cells and cell debris in the pellet were discarded and the virus containing supernatant was harvested and dispensed in 1 ml aliquots. The viral titers were determined by plaque assay as described before (Bidawid et al. 2000, 2003). The viral stocks for both HAV and FCV were 1 × 10^6^ PFU/ml and were stored at −80 °C until use.

### Sample Processing and Virus Precipitation

A 25 g portion of each pork product was added to a sterile 250 mL centrifugation bottle and a subset (13 pâtés, 21 sausages, and 11 livers) of samples was spiked with 5.6 × 10^7^ genome copies FCV as the internal sample process control. Three pork liver samples were spiked with 5.6 × 10^5^ genome copies of HAV. The samples were homogenized using a PRO2-20200 probe (Diamed, Mississauga, Ontario, Canada) in 225 mL of glycine buffer (pH 9.5) for 1 min and placed on ice. Additional homogenization was performed for food matrices which required more time to become fully homogeneous. The homogenization probe was disinfected between samples by immersion into undiluted commercial bleach (6% sodium hypochlorite solution, 5 min) followed by immersion into double-distilled water and a final 70% ethanol wash. The probe was allowed to air dry before homogenizing the next sample. The homogenates were filtered using a device made in-house consisting of a modified bottle-top vacuum filtration unit (Millipore, Etobicoke, Ontario, Canada). The original membrane was removed from the Millipore filtration unit and the following layers were added (described from top to bottom): small aquarium rocks, 3 sequential nylon membranes with 125, 55, and 35 µm pore sizes (Industrial Fabrics Corporation, Minneapolis, Minnesota, U.S.A), separated by Plaskolite louver (Home Depot, Ottawa, Ontario, Canada). The nylon membranes were sealed around their perimeter with 100% silicone caulking and allowed to dry between layers. Prior to use, 500 mL of 70% ethanol was passed through the filter, followed by one rinse with 500 mL of sterile double-distilled water. The filtered homogenates were incubated at 37 °C for 30 min and centrifuged at 3700×*g* for 2 h at 4 °C. To precipitate viruses, the supernatants were decanted into a sterile glass bottle, an equal volume of 16% polyethylene glycol was added and then mixed by inversion. The mixture was incubated for 18 ± 2 h at 4 °C. The precipitated viruses were mixed by inversion and collected by centrifugation at 3700×*g* for 14 min at 4 °C.

### Total RNA Extraction and Viral RNA Concentration

Precipitated virus pellets were dissolved in 5 mL of TRI Reagent^®^ (Sigma-Aldrich, Oakville, Ontario Canada) and transferred to a 15 mL centrifugation tube. If pellets were large, they were split into 2 or more tubes each with 5 mL TRI Reagent^®^. To extract total RNA, 1.2 mL of 1-bromo-3-chloropropane (Sigma-Aldrich) was added and mixed by vortexing. After 5 min incubation at room temperature, the samples were centrifuged at 5000×*g* for 24 min at 4 °C. The upper aqueous layers were transferred to a clean 15 mL centrifugation tube, 0.5 volumes of isopropanol were added, the samples were mixed by inversion, and incubated at room temperature for 5 min. RNA was precipitated by centrifugation at 5000×*g* for 5 min at 4 °C and the RNA pellets were washed with 80% ethanol. Subsequent to air-drying, the RNA was resuspended with 500 µL of DNase/RNase-free water. The total RNA was used immediately or stored at −80 °C until use.

Viral poly (A)-RNA was concentrated from 400 µL of total RNA using Dynabeads Oligo (dT)_25_ (Invitrogen, Burlington, Ontario, Canada) following manufacturer’s instructions for purifying mRNA from total RNA. RNA was eluted with 25 µL of DNase/RNase-free water and released from the beads by heating them to 90 °C for 2 min.

### HEV-Positive Control Preparation

Positive control HEV RNA was extracted from swine fecal filtrate obtained from Dr. Alain Houde and Dr. Julie Brassard (Agriculture and Agri-Food Canada, St-Hyacinthe, Quebec). RNA was extracted using QIAamp Viral RNA mini kit (Qiagen, Mississauga, Ontario, Canada) according to manufacturer’s instructions. Positive control HEV RNA was stored at −80 °C until use.

### RT-PCR Detection of HEV

The RT-PCR detection of HEV RNA was performed using Qiagen’s One-Step RT-PCR kit (Qiagen) using a method described previously (Baylis et al. 2011), with slight modifications. The primer sequences, which targeted the ORF2 region of the HEV genome, were the following (5′–3′): HEV ORF2 (forward) GTYATGYTYTGCATACATGGCT and HEVORF2 (reverse) AGCCGACGAAATYAATTCTGTC. The thermal cycling conditions were: 50 °C for 30 min, 95 °C for 15 min, and 45 cycles of (94 °C for 30 s, 49 °C for 30 s, and 72 °C for 1 min). The RT-PCR products of expected size were gel-purified using the QIAquick gel-extraction kit (Qiagen) according to manufacturer’s instructions.

### DNA Sequencing

Gel-purified RT-PCR products were sequenced directly using the BigDye^®^ terminator v 3.1 DNA sequencing kit (Applied Biosystems, Foster City, California) according to manufacturer’s instructions. Fluorophore-labeled reactions were purified using the Wizard^®^MagneSil^®^ Sequencing Reaction Clean-up System (Promega, Madison, Wisconsin). Samples were sequenced in both directions using a 3130×l Genetic Analyzer and DNA sequences were analyzed using Bionumerics (Applied Maths). HEV-positive sequences were determined by querying NCBI BLAST and edited using BioEdit (Ibis Biosciences, Carlsbad, California).

### Quantification of HEV RNA by Droplet Digital PCR (ddPCR)

HEV-positive RNA samples were quantified using the One-Step RT-dd PCR kit for Probes (Bio-Rad Laboratories Ltd., Mississauga, Ontario, Canada) according to manufacturer’s instructions. Primers and probes used to quantify HEV ORF2 were described previously (Jothikumar et al. 2006). The primer sequences were (5′–3′): JV (forward)—GGTGGTTTCTGGGGTGAC and JV (reverse)- AGGGGTTGGTTGGATGAA. The probe sequence was: (FAM)-TGATTCTCAGCCCTTCGC-(BHQ-1). The thermocycling conditions were: 60 °C for 30 min, 95 °C for 5 min, 40 cycles of (94 °C for 30 s (Ramp = 2 °C/sec), 55 °C for 1 min (Ramp = 2 °C/sec), 65 °C for 30 s (Ramp = 2 °C/sec), and 98 °C for 10 min. ddPCR results were analyzed using the QX200™ Droplet Digital system (Bio-Rad Laboratories Ltd.). Briefly, about 20,000 droplets are generated per sample, and data from at least 12, 000 droplets are used in concentration calculations. QuantaSoft software applies Poisson statistics in order to quantify the concentration of RNA and gives a result in copies/μl of the final ddPCR reaction. More details regarding the application of ddPCR in quantification of HEV load can be found in these two recent studies (Martin-Latil et al. 2016; Nicot et al. 2016).

### Determination of Extraction Efficiency and Limit of Quantification

In order to determine the extraction efficiency or the recovery rate, certain sampled pork pâtés, sausages, and livers were spiked with FCV to serve as an internal sample process control virus. Furthermore, in order to use a sample process virus that is natural to the liver tissue, 3 pork livers were spiked with hepatitis A virus (HAV). The recovery rate was calculated after extraction of nucleic acids and quantification by the ddPCR method as the ratio between the number of genome copies of sample process control virus that were recovered to the number of genome copies used to spike the samples with. Overall the recovery rates in the studied samples varied from 0.06 to 14.36%, with the majority of samples (77% of pork pâtés, 86% of sausages, and 100% of raw pork livers) obtained a recovery rate of 1% or higher, which is consistent with the recovery rates obtained by others in similar food matrices (Berto et al. 2013; Di Bartolo et al. 2012, 2015; Szabo et al. 2015, Wilhelm et al. 2014). The recovery rates obtained from 3 pork livers that were spiked with HAV were 4.4, 4.6, and 7.9%.

In order to assess the limit of detection and limit of quantification of the method used in this study, we inoculated 25 g of HEV-negative pâte samples with 1000 PFU (equivalent to 3130 genome copies as was determined by ddPCR), 500 PFU, and 100 PFU of FCV in triplicate and performed virus recovery using the method described above. The average recovered FCV loads for samples that were spiked with 1000 PFU (3130 genome copies) and 500 PFU of FCV were 393 ± 53 and 220 ± 40 genome copies, respectively. However, we did not detect any FCV RNA in samples that were inoculated with 100 PFU of FCV. Therefore, it can be estimated that the limit of quantification of our method for FCV is lower than 500 PFU and higher than 100 PFU.

### Multiple Sequence Alignments and Phylogenetic Tree Analysis

Multiple sequence alignments were performed using both the Multiple Sequence Comparison by Log-Expectation (MUSCLE) (Edgar 2004) and Clustal W (Larkin et al. 2007). A phylogenetic analysis was performed using the neighbor-joining method based on the 300 nucleotides from the 5′ end of the ORF2 with 1000 bootstrap replicates using the MEGA6 software (Tamura et al. 2013). The sequences obtained in this study have been deposited in GenBank under Accession Numbers KX530971 to KX530999.

## Results

### Identification and Quantification of HEV Isolates

In order to determine the prevalence of HEV in retail pork products marketed in Canada, we screened meal-size (25 g) portions of 76 pork pâtés and 35 raw pork sausages, obtained from six different companies, as well as 19 pork livers purchased from local butchers. Samples that yielded amplicons with expected size (348 bp) were gel-purified and sequenced in both directions to confirm the identity and presence of HEV RNA. Overall, we detected the HEV genome in 36 pâtés (47%) and 2 pork livers (10.5%). We did not detect any HEV genome in the screened raw pork sausages (Table 1). All of the identified HEV genomes belong to genotype 3 (GenBank Accession Numbers KX530971 to KX530999). Also, positive samples were found in all 6 of the screened brands with brand F having the highest number of HEV positives (total of 13) (Table 2).

**Table 1:** Summary of HEV detection in Canadian retail pork products sampled in this study, including the total number of sampled foods, confirmed positives, as well as the HEV RNA concentration demonstrated as genome copies per gram (gc/g)

Food type	Total no. samples	No. HEV-positive samples	Average recovery rate (%)	Range of recovery rates (%)	HEV RNA concentration (gc/g)
Pork pâté	76	36	3.34 (FCV)	0.5–13.5	3.7–500
Raw sausages (pork)	35	0	2.63 (FCV)	0.3–9.0	N/A
Pork livers	19	2	5.45 (FCV) 5.63 (HAV)	1.0–12.3 4.4–7.9	20–40

*FCV* feline calicivirus, *HAV* hepatitis A, *SD* standard deviation, *N*/*A* not applicable

**Table 2:** HEV-positive samples identified in screened pâtés including the manufacturers, expiry dates, and viral RNA concentrations (genome copies per gram (gc/g))

Sample ID	Company	Expiry date	HEV RNA concentration (gc/g)
HEV-2014-007	A	2014-04-04	NQ
HEV-2014-008	B	2014-04-09	16.5
HEV-2014-012	F	2014-04-16	32
HEV-2014-018	C	2014-03-28	28
HEV-2014-014	E	2014-06-24	14
HEV-2014-022	C	2014-04-18	21
HEV-2014-024	C	2014-03-28	11
HEV-2014-028	F	2014-05-27	23
HEV-2014-030	B	2014-06-13	84
HEV-2014-033	B	2014-06-13	13.5
HEV-2014-034	C	2014-04-28	19
HEV-2014-035	C	2014-04-28	26
HEV-2014-036	C	2014-04-28	12.5
HEV-2014-037	C	2014-04-28	21
HEV-2014-038	C	2014-04-28	50
HEV-2014-039	F	2014-05-28	30
HEV-2014-040	F	2014-05-28	311
HEV-2014-043	F	2014-07-22	206
HEV-2014-044	F	2014-07-23	NQ
HEV-2014-046	D	2014-08-15	11
HEV-2014-047	F	2014-07-04	500
HEV-2014-048	E	2014-09-14	25.5
HEV-2014-051	D	2014-08-24	32
HEV-2014-054	F	2014-07-19	28
HEV-2014-057	E	2014-09-19	7.5
HEV-2014-060	F	2014-09-24	4.5
HEV-2014-061	F	2014-09-03	NQ
HEV-2014-063	E	2014-11-11	NQ
HEV-2014-064	E	2014-11-16	8
HEV-2014-065	E	2014-08-30	NQ
HEV-2014-069	F	2014-08-28	6
HEV-2014-073	E	N/A	3.7
HEV-2014-074	E	2014-12-03	14.5
HEV-2014-094	F	2014-12-03	110
HEV-2014-096	F	2014-12-03	9
HEV-2014-097	D	2015-01-10	20

*NQ* not quantifiable

The presence of the HEV genome in the studied samples was further validated and quantified using droplet digital PCR (ddPCR) method (Table 2). The viral load within the HEV-contaminated pork pâtés ranged from 3.7 to 500 genome copies per g. The viral load detected in HEV-positive raw pig livers was 20.7 and 40 genome copies per g, for samples 2015-121 and 2015-123, respectively. No amplification was detected in any of the screened sausages, thus no viral load was obtained for these samples.

### Phylogenetic Analysis

**Fig. 1:**
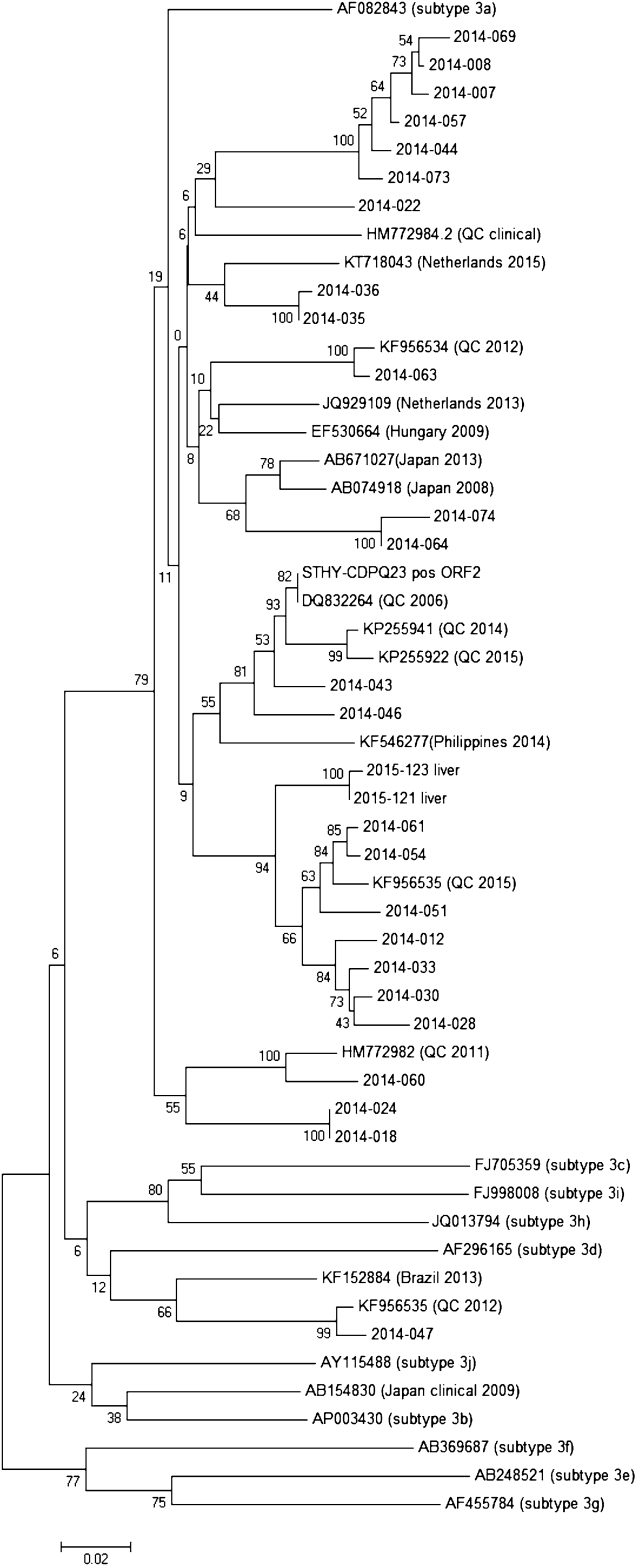
Phylogenetic tree constructed by the neighbor-joining method, based on the 300 nucleotides at the 5′ end of ORF2 gene of the identified isolates in this study as well as closely related strains isolated from swine and human patients. The subtypes of the reference genomes (Smith et al. 2014) are shown in parenthesis. The robustness of the phylogenetic analysis was assessed through bootstrap analysis of 1000 pseudo-replicates. The *scale bar* represents 2% sequence divergence. Each entry is identified with its GenBank Accession Number or the isolate name, as well as the region from which it was isolated. QC province of Quebec, Canada

The amplicons that yielded 300nt sequences from the capsid region (ORF2) of the HEV-positive samples obtained in this study, and highly similar sequences from the NCBI database were aligned and a phylogenetic tree was constructed from this alignment (Fig. 1). Phylogenetic analysis demonstrates that most of the strains isolated in this study belong to genotype 3a and confirmed the relationship between the HEV genomes detected here with the swine HEV strains reported in Canada and around the world. Also, the positive control used in this study, STHY-CDPQ23 pos ORF2 that was isolated from swine fecal matter in the province of Quebec in Canada, clustered with other swine isolates from this region (DQ832264) (100% sequence identity) (Fig. 1). Phylogenetic analysis placed both pork liver HEV isolates, which only differ by one nucleotide, in the same cluster as several other locally acquired HEV isolates from the positive pâtés and another swine isolate from the province of Quebec (KF955635). Moreover, samples 2014-064 and 2014-074 clustered with two Japanese isolates (AB671027, and AB074918), and several other HEV isolates showed sequence similarity with the European isolates. The sequence belonging to sample 2014-047, which had the highest viral load (Table 2), clustered with another isolate from the province of Quebec (KF956535), and is the only isolate in this study that does not belong to subtype 3a (Fig. 1). Importantly, some of the isolates, such as 2014-022 and 2014-060, demonstrated high genetic identity (94 and 97.7%, respectively) with the clinical strains reported in patients in Quebec.

### Analysis of Non-synonymous Variants in the Capsid

**Fig. 2:**
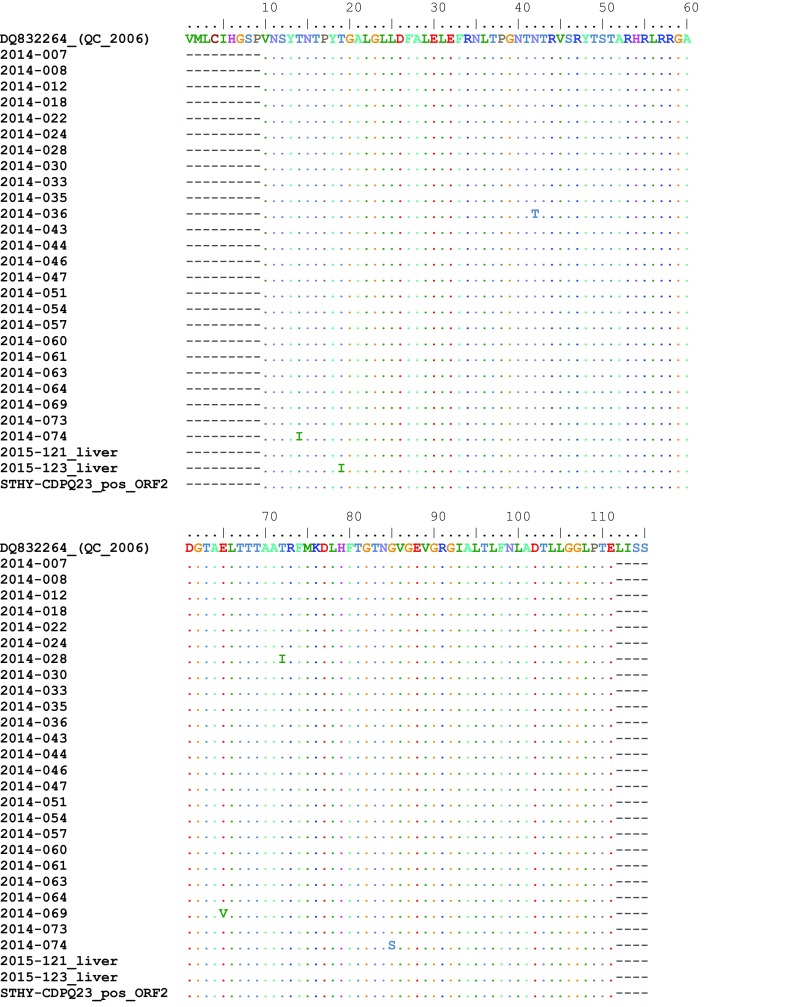
Non-synonymous differences within 100 amino acid sequence of the N domain of the capsid protein (ORF2). Residues are numbered according to a Quebec isolate (Accession No: DQ832264)

The capsid protein contains an N-terminal (N), shell (S), middle (M), and protruding (P) domains (Guu et al. 2009; Kobayashi et al. 2016). The N and S domains are more conserved across HEV strains compared to the P domain. Also the N domain contains several N-linked glycosylation sites (Guu et al. 2009). Herein, 100 amino acids within the N domain of the capsid protein of the isolates were aligned and compared to a reference sequence isolated from Quebec, Canada (Accession No. DQ832264). As shown in Fig. 2, the amino acid sequence of this region is highly conserved between the isolates. There are few biochemically different amino acid substitutions such as N42T in 2014-036, E65 V in 2014-069, G85S in 2014-074, as well as several T/I substitutions in 2014-028, 2014-074, and 2015-123. These amino acid differences could potentially influence the capsid structure, antigenicity, and glycosylation. However, further investigations are necessary to confirm this hypothesis.

## Discussion

In the absence of a robust cell culture system for HEV, molecular detection techniques such as qRT-PCR and ddPCR can be employed to provide useful information on HEV prevalence in foods. To this end, we tested meal-size portions (25 g) of 130 retail pork products including pâtés, sausages, and livers, and identified 38 positive samples overall (Table 1). In addition to conventional RT-PCR, we have used ddPCR, gel purification, and Sanger sequencing to confirm the presence of the HEV genome in the positive samples. Sequencing of the partial capsid region of the positive samples revealed that all isolates belong to genotype 3.

The prevalence of HEV in pâtés in this study (47%) is in agreement with the current HEV-3 distribution in pork products in other developed countries (Berto et al. 2013; Di Bartolo et al. 2012, 2015; Szabo et al. 2015; Wilhelm et al. 2014), and represents the overall HEV infection in the swine herds used for manufacturing pâtés. Failure to detect HEV RNA in the screened sausages may be due to a number of factors including low amount of liver in the making of the sausages, low virus recovery rates, varying amounts of fat and salt concentrations, and/or to the food processing procedures. In fact, the observation that manufacturing procedures can impact the level of HEV contamination and detection has been reported by other investigators as well (Heldt et al. 2016; Szabo et al. 2015). The lower detection of HEV genome in the sampled liver compared to pâtés may be due to several reasons including non-homogenous HEV contamination in the liver, considering the evidence that HEV infection in liver is focal (Di Bartolo et al. 2015). Therefore, it is possible that the liver portions tested in this study were sampled from a non-contaminated section of the liver thereby causing false-negative results. Importantly, it should be taken into account that more than one liver can be present in the same pâté, which might lead to higher prevalence of HEV genome in pâtés (Barnaud et al. 2012). Moreover, HEV contamination of pâtés can occur during production and manufacturing network and might indicate improper food processing during the manufacturing of this product (Nantel-Fortier et al. 2016). The overall HEV detection rate in pork liver reported in this study (10.5%) is in agreement with previous observations (9%) (Nantel-Fortier et al. 2016; Wilhelm et al. 2014).

Proper assessment of molecular quantitative data should take into account the RNA recovery rates for the process control virus. As shown using FCV and HAV as process control viruses in this study, as well as previous reports, the RNA extraction efficiency can largely be influenced by the matrix investigated and the specific extraction method employed (Martin-Latil et al. 2014). In this study, the majority of positive samples yielded acceptable (R > 1%) recovery rates for FCV as the process control virus (Szabo et al. 2015). Also, we employed HAV as the second process control virus for several of the sampled livers, because HAV is a natural pathogen of hepatocytes, which are the main host cells for HEV infection. Herein, the average recovery rate obtained from HAV spiked pork livers (5.63%) was very close to the average recovery rate obtained from FCV pork livers (5.45%), therefore, HAV can potentially be an alternative surrogate for HEV studies in liver matrices.

All the strains detected in this study belong to genotype 3 and were closely related to swine strains detected in Canada, Europe, and Japan. This is not surprising because the international trade of animal and meat commodities makes global circulation of HEV strains possible. The phylogenetic homology between some of our isolates and local clinical isolates may indicate common ancestry, however, for source attribution purposes, more comprehensive analysis such as whole genome sequencing (WGS), and detailed epidemiological data would be necessary (Ronholm et al. 2016).

Although there is significant correlation between genetic and protein evolution, synonymous nucleotide mutations do not result in protein changes, therefore protein evolution is more muted than genetic evolution. Consequently, genetic changes can occur without causing evolutionary effects on viruses (Smith et al. 2004). Herein we monitored the coding differences in the sequenced regions of the isolated HEV strains to determine the functional consequences of the existing genetic variations (Fig. 2). As expected, we identified few amino acid differences between the isolates. Whether these changes alter the structure or antigenicity of the capsid protein is not clear at this point, and in-depth analyses are required to shed more light on this matter.

In this study, we reported on the presence of HEV RNA, which does not provide information on virus infectivity. Thus, it cannot be ruled out that at least in a proportion of the pork products where HEV RNA was detected, the virus was non-infectious. The HEV infectious dose for human is not well established, and it is possible that the low viral load detected in some of the tested samples may or may not contain infectious virus. On the other hand, previous animal models and cell culture studies confirmed the presence of infectious HEV in contaminated commercial pork liver and demonstrated HEV resistance to some of the conventional heat treatments used in the food industry (Barnaud et al. 2012; Berto et al. 2013; Feagins et al. 2008). This is especially important with regards to the products investigated in this study, as it has recently been reported that consuming contaminated pork pâtés and sausages led to an outbreak in Australia (Yapa et al. 2016). It is also of great concern that HEV isolates identified in this and previous studies conducted in Canada, show high homology to clinical isolates detected in this country (Wilhelm et al. 2015a) (Fig. 1), which may suggest potential zoonotic transmission of autochthonous HEV strains.

It is notable that the number of reported cases for human HEV infection is increasing (Blasco-Perrin et al. 2016; Park et al. 2016), which might lead to increased genetic variation and emergence of more virulent strains. Therefore, it is important to monitor and understand the molecular characteristics of circulating HEV strains, and strengthen the source attribution between HEV and causative food products using tools such as WGS. These advancements would support exposure assessment and aid in the determination of health risks and completion of risk assessment.

## Electronic Supplementary Material

Below is the link to the electronic supplementary material.

Supplementary material 1 (DOCX 23 kb)
