# Supplementary material for: Prevalence and Molecular Characterization of the Hepatitis E Virus in Retail Pork Products Marketed in Canada
Source: Food Environ Virol. 2017 Feb 14;9(2):208–18. doi: 10.1007/s12560-017-9281-9 (PMC5429394; doi:10.1007/s12560-017-9281-9)
Supplement: Supplementary file 1 — Supplementary material 1 (DOCX 23 kb) [file 12560_2017_9281_MOESM1_ESM.docx]

| **Sample ID** | **Ingredients** |
| --- | --- |
| HEV-2014-001 | Pork liver, pork, bacon (pork, water, salt, potassium chloride, sugar, sodium phosphate, dextrose, sodium erythorbate, sodium nitrite, spice, smoke), water, wheat flour, salt, corn syrup, solids, spice, onion powder, sodium nitrite. |
| HEV-2014-002 | Pork liver, pork, bacon (pork, water, salt, potassium chloride, sugar, sodium phosphate, dextrose, sodium erythorbate, sodium nitrite, spice, smoke), water, wheat flour, salt, corn syrup, solids, spice, onion powder, sodium nitrite. |
| HEV-2014-003 | Pork liver, pork, bacon (pork, water, salt, potassium chloride, sugar, sodium phosphate, dextrose, sodium erythorbate, sodium nitrite, spice, smoke), water, wheat flour, salt, corn syrup, solids, spice, onion powder, sodium nitrite. |
| HEV-2014-004 | Pork fat, pork liver, pork rind, water, liquid egg-white, modified milk ingredients, onions, white vinegar, salt, pork, spices & herbs, dehydrated garlic, sugar, carrots, celery, sodium erythorbate, sodium nitrite. |
| HEV-2014-005 | Pork fat, pork liver, glaze (water, gelatin, salt, sugar, sodium erythorbate, caramel colour), water, cooked ham, modified milk ingredients, liquid egg white, mushrooms, onions, salt, spices, parsley, garlic, sugar, sodium, erythorbate, sodium nitrite. |
| HEV-2014-006 | Chicken and pork livers, onions, pork fat, water, port wine, modified milk ingredients, dehydrated onions, modified corn starch, wheat protein (gluten), salt, gelatin, brown sugar, pork, seasonings, egg white, garlic, sugar, sodium erythorbate, citric acid, sodium nitrite. |
| HEV-2014-007 | Pork fat, pork liver, frozen egg white |
| HEV-2014-008 | Pork fat, pork liver, pork rind, water, egg white, onions, modified milk ingredients, tapioca starch, salt, pork, sugar, spices, dehydrated garlic, carrots, celery, sodium erythorbate, sodium nitrite. |
| HEV-2014-009 | Pork, water, toasted wheat crumbs, dehydrated onions, salt, spices. |
| HEV-2014-010 | Pork fat, pork liver, pork rind, ham (pork, water, glucose, salt, flavour), etc. |
| HEV-2014-011 | Pork fat, pork liver pork, pork rind, water, liquid egg-white, modified milk ingredients, onions, salt, pork, spices& herbs, etc. |
| HEV-2014-012 | Pork and chicken liver, pork fat, pork, onions, modified milk ingredients, modified corn starch, white vinegar, salt, cognac, spices, dehydrated garlic, sodium erythorbate, liquid egg-white, sodium nitrite. |
| HEV-2014-013 | Pork, pork liver, glaze (water, gelatin, salt, dextrose, port wine, red currant syrup, black pepper, red and green pepper, orange zest, sodium erythorbate), modified milk ingredients, water, onions, liquid egg white, salt, spices, dextrose, parsley, garlic sodium erythorbate, grand-marnier liqueur, sodium nitrite. |
| HEV-2014-014 | Pork, pork liver, water, beef fat, egg albumen, pork skin, potassium lactate, turkey, salt, onion powder, modified milk ingredients, spices, wheat flour, hydrolysed soy protein, mustard seeds, dextrose, sodium dioxide, sodium erythorbate, sodium nitrite. |
| HEV-2014-015 | Pork, pork liver, water, modified potato starch, salt, sodium citrate, onion powder, sodium ascorbate, spices and spice extractives, ascorbic acid, flavour, sodium nitrite. |
| HEV-2014-016 | Pork, pork liver, water, modified potato starch, salt, sodium citrate, onion powder, sodium ascorbate, spices and spice extractives, ascorbic acid, flavour, sodium nitrite. |
| HEV-2014-017 | Pork, pork liver, water, beef fat, egg albumen, pork skin, potassium lactate, turkey, salt, onion powder, modified milk ingredients, spices, wheat flour, hydrolysed soy protein, mustard seeds, dextrose, sodium dioxide, sodium erythorbate, sodium nitrite. |
| HEV-2014-018 | Pork, pork liver, water, modified corn starch, salt, etc. |
| HEV-2014-022 | Pork, pork liver, water, modified corn starch, salt, etc. |
| HEV-2014-023 | Pork, pork liver, water, modified corn starch, salt, etc. |
| HEV-2014-024 | Pork, pork liver, water, modified corn starch, salt, etc. |
| HEV-2014-026 | Pork fat, pork liver pork, pork rind, water, liquid egg-white, modified milk ingredients, onions, salt, pork, spices& herbs, etc. |
| HEV-2014-027 | Pork fat, pork liver, pork rind, water, liquid egg-white, modified milk ingredients, onions, white vinegar, salt, pork, spices & herbs, dehydrated garlic, sugar, carrots, celery, sodium erythorbate, sodium nitrite. |
| HEV-2014-028 | Pork fat, pork liver, pork rind, ham (pork, water, glucose, salt, flavour), etc. |
| HEV-2014-029 | Pork, pork liver, onions, chicken liver, modified milk ingredients, whole black peppercorns, salt, spices, modified corn starch, malt vinegar, dehydrated garlic, pepper sauce (white vinegar, hot red pepper, salt), sodium erythorbate, sodium nitrite. |
| HEV-2014-030 | Pork fat, pork liver, water, pork, pork rind, onions, egg white, salt, modified milk ingredients, tapioca starch, modified corn starch, dehydrated vegetables (green peas, green and red bell peppers), spices, sugar, sodium phosphate, celery, carrots, dehydrated garlic, sodium erythorbate, sodium nitrite. |
| HEV-2014-031 | Pork fat, pork liver, water, pork, pork rind, onions, egg white, salt, modified milk ingredients, tapioca starch, modified corn starch, spices, cognac, sugar, sodium phosphate, celery, carrots, dehydrated garlic, sodium erythorbate, sodium nitrite. |
| HEV-2014-032 | Pork fat, pork liver, water, pork, pork rind, onions, egg white, salt, modified milk ingredients, tapioca starch, modified corn starch, spices, sugar, sodium phosphate, carrots, celery, dehydrated garlic, sodium erythorbate, sodium nitrite. |
| HEV-2014-033 | Pork fat, pork liver, water, pork, pork rind, onions, egg white, salt, modified milk ingredients, tapioca starch, spices, modified corn starch, sugar, sodium phosphate, celery, carrots, dehydrated garlic, sodium erythorbate, sodium nitrite. |
| HEV-2014-034 | Pork liver, pork, bacon (pork, water, salt, potassium chloride, sugar, sodium phosphate, dextrose, sodium erythorbate, sodium nitrite, spice, smoke), water, wheat flour, salt, corn syrup, solids, spice, onion powder, sodium nitrite. |
| HEV-2014-035 | Pork liver, pork, bacon (pork, water, salt, potassium chloride, sugar, sodium phosphate, dextrose, sodium erythorbate, sodium nitrite, spice, smoke), water, wheat flour, salt, corn syrup, solids, spice, onion powder, sodium nitrite. |
| HEV-2014-036 | Pork liver, pork, bacon (pork, water, salt, potassium chloride, sugar, sodium phosphate, dextrose, sodium erythorbate, sodium nitrite, spice, smoke), water, wheat flour, salt, corn syrup, solids, spice, onion powder, sodium nitrite. |
| HEV-2014-037 | Pork liver, pork, bacon (pork, water, salt, potassium chloride, sugar, sodium phosphate, dextrose, sodium erythorbate, sodium nitrite, spice, smoke), water, wheat flour, salt, corn syrup, solids, spice, onion powder, sodium nitrite. |
| HEV-2014-038 | Pork liver, pork, bacon (pork, water, salt, potassium chloride, sugar, sodium phosphate, dextrose, sodium erythorbate, sodium nitrite, spice, smoke), water, wheat flour, salt, corn syrup, solids, spice, onion powder, sodium nitrite. |
| HEV-2014-039 | Pork and chicken liver, pork fat, pork, onions, modified milk ingredients, modified corn starch, white vinegar, salt, cognac, spices, dehydrated garlic, sodium erythorbate, liquid egg-white, sodium nitrite. |
| HEV-2014-040 | Pork and chicken liver, pork fat, pork, onions, modified milk ingredients, modified corn starch, white vinegar, salt, cognac, spices, dehydrated garlic, sodium erythorbate, liquid egg-white, sodium nitrite. |
| HEV-2014-041 | Pork, pork liver, glaze (water, gelatin, salt, dextrose, port wine, red currant syrup, black pepper, red and green pepper, orange zest, sodium erythorbate), modified milk ingredients, water, onions, liquid egg white, salt, spices, dextrose, parsley, garlic sodium erythorbate, grand-marnier liqueur, sodium nitrite. |
| HEV-2014-042 | Pork fat, pork liver, glaze (water, gelatin, salt, sugar, sodium erythorbate, caramel colour), water, cooked ham, modified milk ingredients, liquid egg white, mushrooms, onions, salt, spices, parsley, garlic, sugar, sodium, erythorbate, sodium nitrite. |
| HEV-2014-043 | Pork fat, pork liver, pork rind, ham (pork, water, glucose, salt, flavour), etc. |
| HEV-2014-044 | Pork and chicken liver, pork fat, pork, onions, modified milk ingredients, modified corn starch, white vinegar, salt, cognac, spices, dehydrated garlic, sodium erythorbate, liquid egg-white, sodium nitrite. |
| HEV-2014-045 | Pork fat, pork liver, glaze (water, gelatin, salt, sugar, sodium erythorbate, caramel colour), water, cooked ham, modified milk ingredients, liquid egg white, mushrooms, onions, salt, spices, parsley, garlic, sugar, sodium, erythorbate, sodium nitrite. |
| HEV-2014-046 | Pork, pork liver, water, modified potato starch, salt, sodium citrate, onion powder, sodium ascorbate, spices and spice extractives, ascorbic acid, flavour, sodium nitrite. |
| HEV-2014-047 | Pork, pork liver, glaze (water, gelatin, salt, dextrose, port wine, red currant syrup, black pepper, red and green pepper, orange zest, sodium erythorbate), modified milk ingredients, water, onions, liquid egg white, salt, spices, dextrose, parsley, garlic sodium erythorbate, grand-marnier liqueur, sodium nitrite. |
| HEV-2014-048 | Pork, pork liver, water, beef fat, egg albumen, pork skin, potassium lactate, turkey, salt, onion powder, modified milk ingredients, spices, wheat flour, hydrolysed soy protein, mustard seeds, dextrose, sodium dioxide, sodium erythorbate, sodium nitrite. |
| HEV-2014-049 | Pork fat, pork liver pork, pork rind, water, liquid egg-white, modified milk ingredients, onions, salt, pork, spices& herbs, etc. |
| HEV-2014-050 | Pork, pork liver, water, modified potato starch, salt, sodium citrate, herbs, spices and spice extracts, onion powder, sodium ascorbate, ascorbic acid, flavour, sodium nitrite. |
| HEV-2014-051 | Pork, pork liver, water, modified potato starch, salt, sodium citrate, onion powder, sodium ascorbate, spice extractives, ascorbic acid, flavour, sodium nitrite. |
| HEV-2014-052 | Pork fat, pork liver, pork rind, ham (pork, water, glucose, salt, flavour), etc. |
| HEV-2014-053 | Pork fat, pork liver, glaze (water, gelatin, salt, sugar, sodium erythorbate, caramel colour), water, cooked ham, modified milk ingredients, liquid egg white, mushrooms, onions, salt, spices, parsley, garlic, sugar, sodium, erythorbate, sodium nitrite. |
| HEV-2014-054 | Pork, pork liver, glaze (water, gelatin, salt, dextrose, port wine, red currant syrup, black pepper, red and green pepper, orange zest, sodium erythorbate), modified milk ingredients, water, onions, liquid egg white, salt, spices, dextrose, parsley, garlic sodium erythorbate, grand-marnier liqueur, sodium nitrite. |
| HEV-2014-055 | Pork fat, pork liver, pork rind, water, liquid egg-white, modified milk ingredients, onions, white vinegar, salt, pork, spices & herbs, dehydrated garlic, sugar, carrots, celery, sodium erythorbate, sodium nitrite. |
| HEV-2014-056 | Pork and chicken liver, pork fat, pork, onions, modified milk ingredients, modified corn starch, white vinegar, salt, cognac, spices, dehydrated garlic, sodium erythorbate, liquid egg-white, sodium nitrite. |
| HEV-2014-057 | Pork, pork liver, water, beef fat, egg albumen, pork skin, potassium lactate, turkey, salt, onion powder, modified milk ingredients, spices, wheat flour, hydrolysed soy protein, mustard seeds, dextrose, sodium dioxide, sodium erythorbate, sodium nitrite. |
| HEV-2014-058 | Pork, water, toasted wheat crumbs, dehydrated onions, salt, spices. |
| HEV-2014-059 | Pork, pork liver, onions, chicken liver, modified milk ingredients, whole black peppercorns, salt, spices, modified corn starch, malt vinegar, dehydrated garlic, pepper sauce (white vinegar, hot red pepper, salt), sodium erythorbate, sodium nitrite. |
| HEV-2014-060 | Pork fat, pork liver pork, pork rind, water, liquid egg-white, modified milk ingredients, onions, salt, pork, spices& herbs, etc. |
| HEV-2014-061 | Pork and chicken liver, pork fat, pork, onions, modified milk ingredients, modified corn starch, white vinegar, salt, cognac, spices, dehydrated garlic, sodium erythorbate, liquid egg-white, sodium nitrite. |
| HEV-2014-062 | Pork fat, pork liver, pork rind, water, liquid egg-white, modified milk ingredients, onions, white vinegar, salt, pork, spices & herbs, dehydrated garlic, sugar, carrots, celery, sodium erythorbate, sodium nitrite. |
| HEV-2014-063 | Pork, pork liver, water , beef fat, egg albumen, turkey, salt, red bell peppers, onion powder, modified milk ingredients, spices, herbs, dextrose, sodium erythorbate, sodium nitrite. |
| HEV-2014-064 | Pork, pork liver, water, beef fat, egg albumen, pork skin, potassium lactate, turkey, salt, onion powder, modified milk ingredients, spices, wheat flour, hydrolysed soy protein, mustard seeds, dextrose, sodium dioxide, sodium erythorbate, sodium nitrite. |
| HEV-2014-065 | Pork, pork liver, water, beef fat, egg albumen, pork skin, potassium lactate, turkey, salt, onion powder, modified milk ingredients, spices, wheat flour, hydrolysed soy protein, mustard seeds, dextrose, sodium dioxide, sodium erythorbate, sodium nitrite. |
| HEV-2014-066 | Pork fat, pork liver pork, pork rind, water, liquid egg-white, modified milk ingredients, onions, salt, pork, spices& herbs, etc. |
| HEV-2014-067 | Pork, pork liver, onions, chicken liver, modified milk ingredients, whole black peppercorns, salt, spices, modified corn starch, malt vinegar, dehydrated garlic, pepper sauce (white vinegar, hot red pepper, salt), sodium erythorbate, sodium nitrite. |
| HEV-2014-068 | Pork fat, pork liver, glaze (water, gelatin, salt, sugar, sodium erythorbate, caramel colour), water, cooked ham, modified milk ingredients, liquid egg white, mushrooms, onions, salt, spices, parsley, garlic, sugar, sodium, erythorbate, sodium nitrite. |
| HEV-2014-069 | Pork, pork liver, glaze (water, gelatin, salt, dextrose, port wine, red currant syrup, black pepper, red and green pepper, orange zest, sodium erythorbate), modified milk ingredients, water, onions, liquid egg white, salt, spices, dextrose, parsley, garlic sodium erythorbate, grand-marnier liqueur, sodium nitrite. |
| HEV-2014-070 | Pork, water, toasted wheat crumbs, dehydrated onions, salt spices. |
| HEV-2014-071 | Pork, pork liver, water, modified potato starch, salt, sodium citrate, onion powder, sodium ascorbate, spices and spice extractives, ascorbic acid, flavour, sodium nitrite. |
| HEV-2014-072 | Pork, pork liver, water, modified potato starch, salt, sodium citrate, herbs, spices and spice extracts, onion powder, sodium ascorbate, ascorbic acid, flavour, sodium nitrite. |
| HEV-2014-073 | Pork, pork liver, water, beef fat, egg albumen, pork skin, potassium lactate, turkey, salt, onion powder, modified milk ingredients, spices, wheat flour, hydrolysed soy protein, mustard seeds, dextrose, sodium dioxide, sodium erythorbate, sodium nitrite. |
| HEV-2014-074 | Pork, pork liver, water , beef fat, egg albumen, turkey, salt, red bell peppers, onion powder, modified milk ingredients, spices, herbs, dextrose, sodium erythorbate, sodium nitrite. |
| HEV-2014-075 | Pork, water, honey, cane sugar, sea salt, dried vinegar, dehydrated garlic, spice. |
| HEV-2014-076 | Pork, water, toasted wheat crumbs, sea salt, seasoning, sugar. In collagen casing. |
| HEV-2014-077 | Pork, water, sea salt, sugar, seasonings, potassium chloride, garlic extract, toasted wheat crumbs, pork natural casing. |
| HEV-2014-078 | Pork, water, toasted wheat crumbs, oat hull powder (contains salt, flavour, soybean oil), brown sugar, salt, seasoning. |
| HEV-2014-079 | Pork, water, toasted wheat crumbs, sea salt, seasonings, sugar. In collagen casing. |
| HEV-2014-080 | Pork, water, sea salt, sugar, seasonings, potassium chloride, garlic extract, toasted wheat crumbs, pork natural casing. |
| HEV-2014-081 | Pork, water, spice, sea salt, dehydrated red bell pepper, dried vinegar, dehydrated garlic, cane sugar. |
| HEV-2014-082 | Pork, water, sea salt, seasonings, sugar, garlic, toasted wheat crumbs. In natural hog casing. |
| HEV-2014-083 | Pork, water, corn syrup, salt, spices, dextrose, lemon juice powder (corn syrup and lemon juice solids, natural flavours), dehydrated red bell pepper, natural flavours, spice extracts, silicon dioxide. |
| HEV-2014-084 | Pork, water, toasted wheat crumbs, sea salt, seasoning, sugar. In collagen casing. |
| HEV-2014-085 | Pork, brown sugar, water, salt, sugar, corn syrup, seasoning (cellulose gel, cornstarch, maltodextrin, modified cornstarch, molasses, glycerin, sugar dextrose, honey), dextrose, lemon juice powder (maltodextrin, lemon juice solids), monosodium glutamamte, flavours, silicon dioxide, calcium silicate, calcium stearate. |
| HEV-2014-086 | Pork, water, sea salt, seasonings, garlic, sugar, toasted wheat crumbs. In hog natural casing. |
| HEV-2014-087 | Pork, water, toasted wheat crumbs, sea salt, seasonings, salt, natural flavour. In hog natural casing. |
| HEV-2014-088 | Pork, water, sea salt, sugar, seasonings, potassium chloride, garlic extract, toasted wheat crumbs, pork natural casing. |
| HEV-2014-089 | Pork, processed cheddar cheese, water, corn syrup, salt, bacon flavour, natural flavour, silicon dioxide. Contains milk and soy. |
| HEV-2014-090 | Pork, water, honey powder (honey, maltodextrin), sea salt, honey, fructose, sugar, garlic powder, seasonings, toasted wheat crumbs, flavour. In hog natural casing. |
| HEV-2014-091 | Pork, water, maple syrup, sea salt, dextrose, durum wheat flour, brown sugar, seasonings, flavour. In collagen casing. |
| HEV-2014-092 | Pork, brown sugar, water, salt, sugar, corn syrup, seasoning (cellulose gel, cornstarch, maltodextrin, modified cornstarch, molasses, glycerin, sugar dextrose, honey), dextrose, lemon juice powder (maltodextrin, lemon juice solids), monosodium glutamate, flavours, silicon dioxide, calcium silicate, calcium stearate. |
| HEV-2014-093 | Pork, water, corn syrup, salt, dextrose, lemon juice powder (corn syrup and lemon juice solids, natural flavours), spices, natural flavour, paprika extracts, silicon dioxide. |
| HEV-2014-094 | Pork fat, pork liver, pork rind, ham (pork, water, glucose, salt, flavour), etc. |
| HEV-2014-095 | Pork fat, pork liver, pork rind, water, liquid egg-white, modified milk ingredients, onions, white vinegar, salt, pork, spices & herbs, dehydrated garlic, sugar, carrots, celery, sodium erythorbate, sodium nitrite. |
| HEV-2014-096 | Pork fat, pork liver, pork rind, etc. |
| HEV-2014-097 | Pork, pork liver, water, modified potato starch, salt, sodium citrate, onion powder, sodium ascorbate, spices and spice extractives, ascorbic acid, flavour, sodium nitrite. |
| HEV-2014-098 | Pork, water, toasted wheat crumbs, sea salt, seasonings, salt, natural flavour. In hog natural casing. |
| HEV-2014-099 | Pork, water, toasted wheat crumbs, sea salt, seasoning, sugar. In collagen casing. |
| HEV-2014-100 | Pork, water, corn syrup, salt, dextrose, lemon juice powder (maltodextrin, lemon juice solids), monosodium glutamate, flavours, calcium stearate, silicon dioxide. |
| HEV-2014-101 | Pork, brown sugar, water, salt, sugar, corn syrup, seasoning (cellulose gel, cornstarch, maltodextrin, modified cornstarch, molasses, glycerin, sugar dextrose, honey), dextrose, lemon juice powder (maltodextrin, lemon juice solids), monosodium glutamate, flavours, silicon dioxide, calcium silicate, calcium stearate. |
| HEV-2014-102 | Pork, water, sea salt, seasonings, garlic, sugar, toasted wheat crumbs. In hog natural casing. |
| HEV-2014-103 | Pork, water, toasted wheat crumbs, sea salt, seasonings, sugar. In collagen casing. |
| HEV-2014-104 | Pork, water, corn syrup, salt, dextrose, sugar, spices, natural flavours, paprika extracts, silicon dioxide. |
| HEV-2014-105 | Pork, water, corn syrup, salt, spices, dextrose, lemon juice powder (corn syrup and lemon juice solids, natural flavours), dehydrated red bell pepper, natural flavours, spice extracts, silicon dioxide. |
| HEV-2014-106 | Pork, water, corn syrup, salt, dextrose, lemon juice powder (corn syrup and lemon juice solids, natural flavours), spices, natural flavour, paprika extracts, silicon dioxide. |
| HEV-2014-107 | Pork, water, salt, vinegar, spices, dehydrated red bell pepper, sugar, natural flavour. |
| HEV-2014-108 | Pork, water, corn syrup, salt, dextrose, lemon juice powder (maltodextrin, lemon juice solids), monosodium glutamate, flavours, calcium stearate, silicon dioxide. |
| HEV-2014-109 | Pork, water, corn syrup, salt, dextrose, monosodium glutamate, lemon juice powder (corn syrup and lemon juice solids), natural flavour, flavours, calcium silicate, silicon dioxide. |
| HEV-2014-110 | Pork, water, dried vinegar, sea salt, cane sugar, spice. |
| HEV-2014-111 | Pork, water, spice, sea salt, dried vinegar, dehydrated red bell pepper, cane sugar, dehydrated garlic. |
| HEV-2014-112 | Pork, water, maple syrup, maple sugar, sea salt, dried vinegar, spice. |
| HEV-2014-113 | Pork, water, sugar, salt, vinegar, spices, honey and garlic flavours. |
| HEV-2014-114 | Pork, pork liver, milk, white wine (sulfites), liquid eggs (eggs, citric acid), onions, sea salt, spices, modified milk ingredients, soy proteins, soy proteins, sugar, brine (salt, sodium nitrite, sodium bicarbonate, canola oil), sodium erythorbate. |
| HEV-2014-115 | Duck, pork, pork liver, milk, liquid eggs (eggs, citric acid), concentrated orange juice (water, concentrated orange juice), onions, candy orange peel (orange peels, glucose-fructose syrup, sugar, dextrose, citric acid, sulfites), gelified orange extract, sea salt, sugar, modified milk ingredients, soy proteins, spices, brine (salt, sodium nitrite, sodium bicarbonate, canola oil), sodium erythorbate. |
